# Effect of an interactive mobile health support system and daily weight measurements for pediatric obesity treatment, a 1-year pragmatical clinical trial

**DOI:** 10.1038/s41366-022-01146-8

**Published:** 2022-05-31

**Authors:** Emilia Hagman, Linnea Johansson, Claude Kollin, Erik Marcus, Andreas Drangel, Love Marcus, Claude Marcus, Pernilla Danielsson

**Affiliations:** 1 Department of Clinical Science, Intervention and Technology, Division of Pediatrics, Karolinska Institutet, Blickagången 6A, Novum, S-141 57 Huddinge, Sweden; 2 Health Professionals Function, Medical Unit Occupational Therapy & Physiotherapy, Karolinska University Hospital, Hälsovägen 13, S-141 57 Huddinge, Sweden; 3 Martina Children’s Hospital, Valhallavägen 91F, S-114 86 Stockholm, Sweden; 4 Evira AB, Sibyllegatan 28, S-114 43 Stockholm, Sweden

**Keywords:** Paediatrics, Clinical trials

## Abstract

**Background:**

Pediatric obesity lifestyle treatment is not always successful. Frequent clinical visits are of major importance to certify sufficient effect but are difficult due to the associated costs and the great demands on families. We hypothesized that an interactive digital support may reduce the need for frequent physical visits. The aim of the study was to assess 1-year weight outcome for patients using a digital support system compared with standard care.

**Methods:**

An obesity lifestyle treatment with a digital support system was implemented in one clinic in Stockholm, Sweden. Measurements from a custom-made body scale without digits for daily home measurement of weights were transferred via Bluetooth to a mobile application, where BMI *Z*-score was calculated and presented graphically with an individualized weight loss target curve. An automatic transfer of data to the web-based clinic interface enables a close monitoring of treatment progress, and frequent written communication between the clinical staff and families via the application. One-year outcome was compared with a randomly retrieved, age and sex matched control group from the Swedish childhood obesity treatment register (BORIS), which received standard treatment at other clinics. Main outcome was change in BMI *Z*-score and missing data was imputed.

**Results:**

107 children were consecutively included to digi-physical treatment and 321 children to standard care. Age range 4.1–17.4 years (67% males). The attrition rate was 36% and 46% respectively, *p* = 0.08. After 1 year, the mean ± SD change in BMI *Z*-score in the treatment group was −0.30 ± 0.39 BMI *Z*-score units and in the standard care group −0.15 ± 0.28, *p* = 0.0002. The outcome was better for both sexes and all age groups in the digi-physical treated group.

**Conclusion:**

A digital support system with a personalized weight-loss target curve and daily weight measurements shared by the family and the clinic is more effective than a standard care childhood obesity treatment.

**Clinicaltrial.gov ID:**

NCT04323215

## Background

Obesity is a growing global public health concern. Children and adolescents with obesity face several complications including metabolic co-morbidities, psychosocial limitations, and premature mortality [1–4]. Obesity in childhood and adolescence is associated with lower educational achievement, regardless of socio-economic status [5]. All these consequences in childhood seem to be reversible through weight loss [1–5].

It is well established that standard pediatric obesity treatment is ineffective [6, 7]. Most programs demonstrate an initial relative weight loss indicating that the teaching is successful and that most families understand what they have to do. However, weight regain is often observed after the end of the intervention which may indicate that ongoing continuous support is required for most families [6, 7].

There is a correlation between the frequency of visits and weight outcome [8]. The US Preventive Services Task Force estimated that families with children suffering from obesity need at least 26 h of contact with the behavioral support team per year to enable children to reach a clinically relevant reduction in the degree of obesity [9]. Weekly visits were superior to visits every other week, however, frequent physical visits are costly and place great demands on families. It is unclear why frequent visits are important, but we hypothesize that frequent feedback to the families in combination with the opportunity for continuous support based on current weight changes are of major importance. Hence, the family can obtain advice and support on how to handle difficult situations when it is required and not several months later. In adults with obesity, daily self-monitoring of weight has shown positive effects on weight outcome which support this hypothesis [10–12].

Digital tools (mHealth) have potential to reduce costs and physical clinical time. They have grown in popularity in obesity treatment, with several approaches [13, 14]. These studies indicate that mHealth systems allowing accessible interaction, frequent contact, and data-monitoring through mobile applications, personal digital assistants and other devices, may result in even better short-term outcome. Several studies have shown improvements in self-reported diet, physical activity and short-term weight loss, but to the best of our knowledge, the addition of mHealth to clinical support for more than 6 months has so far failed to result in an improved weight outcome in children with obesity [15–21].

We have recently shown good feasibility of this digital support system in a small randomized 6-month study. The results showed that both parents and clinicians had positive experience of the system and found it accessible. The system, now named Evira^®^, was used with daily weight measurements and weight outcome targets [22]. The primary aim of the present study was to use a pragmatic study approach to assess the weight outcomes over a period of 1 year in a childhood obesity clinic that implemented digi-physical treatment with this system compared with matched controls in whom conventional standard lifestyle support was used [23]. The secondary aim was to study the weight outcome among subgroups of digi-physically treated individuals.

## Methods

### Participants and setting

To show the real-world effectiveness of a digital support system in combination with clinical visits in a broad patient group, we have conducted a pragmatical clinical trial [24]. Hence, no extra study visits, procedures, or questionnaires have taken place.

**Fig. 1:**
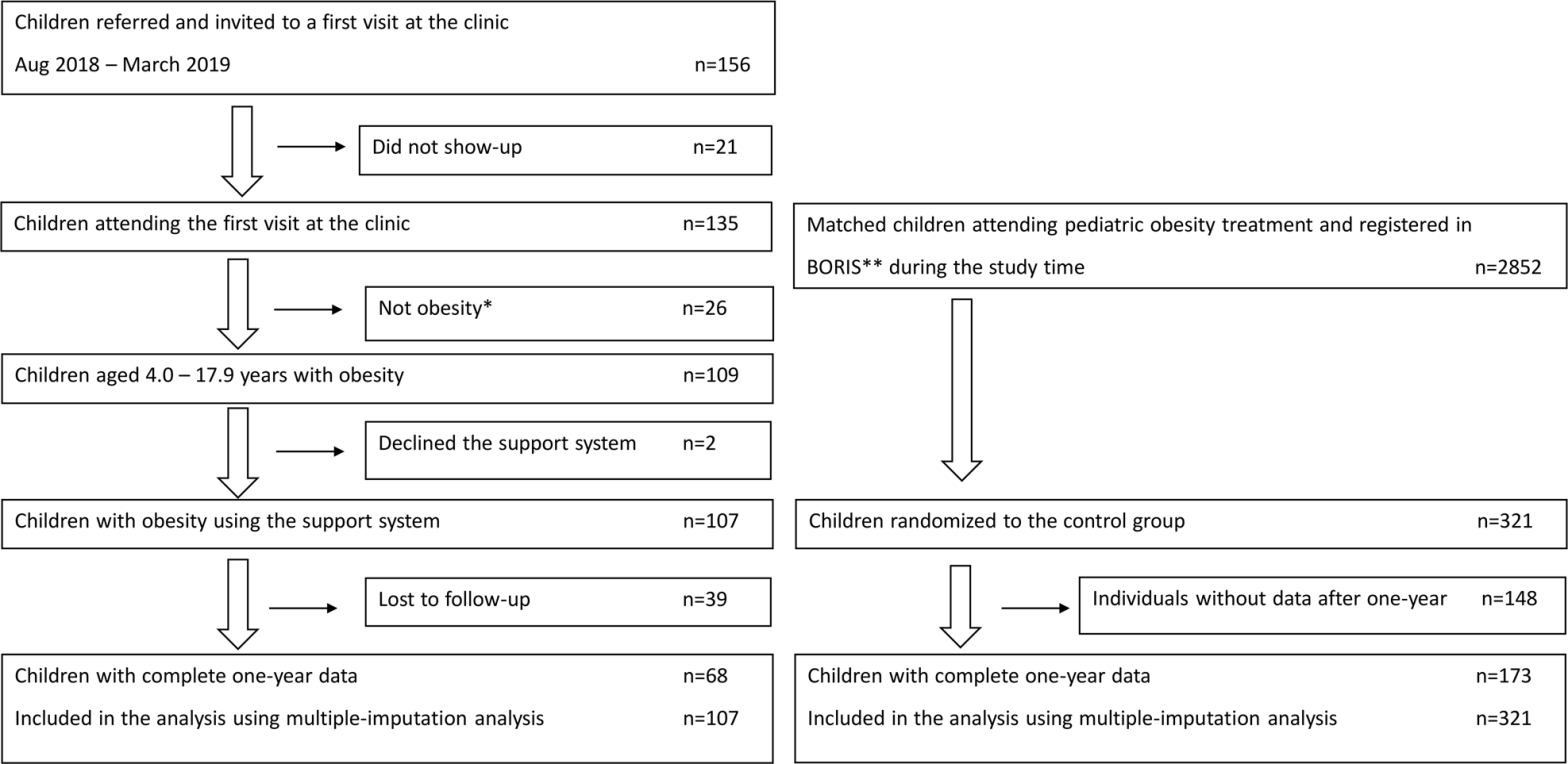
Flowchart of the children treated with digital support and children in standard care. * I.e. patients not meeting the criterion for obesity at treatment initiation. ** BORIS—the Swedish Childhood Obesity Treatment Register.

The inclusion process is described in Fig. 1. Eligible individuals were children and adolescents with obesity between 4.0 and 17.9 years of age, who were referred to Martina Children’s Hospital, Stockholm, Sweden between August 2018 and March 2019 where the weight loss treatment program based on the digital support system was initiated. The obesity clinic was established in August 2018 to provide and evaluate digital support integrated into the treatment of childhood obesity. A multidisciplinary team including pediatricians, pediatric nurses, dieticians, and physiotherapists handled both the physical visits and the digital support. The patients were referred from child healthcare centers, the school health service, and pediatric outpatient clinics in Stockholm County. To obtain a representative case-mix, no restrictions for referral were set in terms of other diseases, neuropsychiatric disorders, or previous obesity treatment. The treatment was provided free of charge as all health care for children is in Sweden, including devices such as the provided body scales.

The control group receiving standard care consisted of individuals from the Swedish Childhood Obesity Treatment Register (BORIS) [23]. The standard care group was matched in a ratio of 3:1, based on sex and age (±91 days) and were randomly selected from 2852 eligible individuals in the BORIS cohort who had their obesity treatment initiated between October 1st, 2017 to January 1st, 2019. In conformity with the digital support group, all controls were diagnosed with obesity [25] and none was to attend tertiary care (University clinics) as they have a different case-mix with more severe obesity and/or co-morbidities and do not match the situation at the intervention clinic. The individuals in the standard care group came from 59 pediatric clinics from different parts of Sweden that provided standard care, namely traditional lifestyle behavioral treatment [23].

According to Swedish regulations, families were informed verbally and in writing about data collection in the BORIS register and at Martina Children’s Hospital.

Post an opt-out approval (possibility to choose not to participate) by parents/guardians, data of the children’s weight and height were recorded by the local healthcare provider during treatment visits. Ethical approval was obtained from the Ethics Committee in Stockholm, Sweden (No. 2018/1413–31) and registered in Clinicaltrail.gov ID: NCT04323215.

### The mHealth support system

**Fig. 2:**
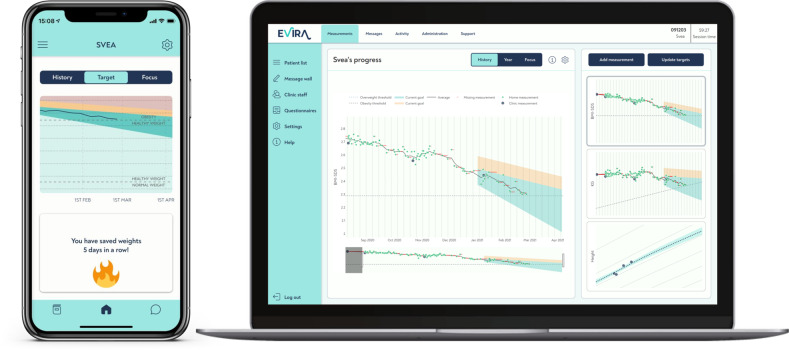
Illustration of the digital support system. Family interface in mobile application shows graphic presentation of child’s individual target curve and progress. Clinic interface shows child’s absolute measures and progress in BMI *Z*-score and weight. Custom made scale, with hidden digits, transfer weight through Bluetooth.

The mHealth support system was developed by Evira AB (Stockholm, Sweden). The treatment with the digital support system was integrated with clinical treatment and based on four cornerstones: (1) a custom-made body scale; (2) a personalized weight development target curve in a mobile application; (3) close monitoring by clinical staff of treatment progress; (4) and frequent communication between the clinical staff and families. The patients accessed the digital support system through a mobile application and the health care professionals accessed the system through a web page (Fig. 2). Objective weight data was used to turn focus of the behavioral support from self-reported process goals towards objectively measured weight-loss goals and ways to achieve them. The digital support system was approved as an EU medical device class 1.

The body scale for home use was connected by Bluetooth to the mobile application which was compatible with Android and iOS devices. To reduce focus on a single weight measurement, the scales did not display digits. Instead, the measured weights in kilograms were recalculated and presented graphically as a weighted moving average of Body Mass Index (BMI) *Z*-score in the mobile application, since absolute weight changes are difficult to interpret in growing subjects. The objective weight data collected in the home setting were then transferred to the clinic via the mobile application. To get a reliable estimate of BMI *Z*-score, the longitudinal growth of the children was estimated by the system in between the height measurements made at the clinic every third month. Since children with obesity have higher growth velocity before puberty and lower during puberty compared with normal weight peers, an obesity-specific predictive longitudinal growth estimation algorithm was constructed taking into consideration the degree of obesity, age and sex. These estimations were based on data from the BORIS [23] (separate ethical approval: 2014/381–31/5) and finely tuned using data from the Evira database. The BMI *Z*-scores presented in the mobile application were calculated based on these longitudinal growth estimations and the children’s daily weight measurements (Supplementary material).

At every 3-month physical follow-up visit, height and weight were measured and the BMI *Z*-score target curve for the forthcoming 3 months was updated. The personalized weight development target curve was displayed in the mobile application and on the clinic’s interface. The target curve range included a maximum and minimum range of recommended future BMI *Z*-score, and the slope of the curve was based on the degree of obesity (Fig. 2). Each curve was automatically created by the system and the maximum and minimum values of change in BMI *Z*-score ranged from −0.15 to −0.35 units over 3 months. Individual adjustments based on age, degree of obesity, metabolic risk estimations and psychosocial status were made by the clinical staff in dialogue with the family. In the clinic interface, staff received the same data as the families, with the addition of absolute weights. If weighing frequency declined, the system signaled the staff. All communication in the system was individualized, i.e., no automatic push notifications were used.

### Implementation of digital support

Before treatment initiation, the child met a pediatrician for a physical check-up, a dietician, and most also met a physiotherapist. A pediatric nurse instructed the family on how to use the digital system. At baseline, the parents and (if applicable) the child downloaded the mobile application together with the nurse and were provided with the custom body scale. Instructions included daily monitoring of the child’s weight. Thereafter physical visits were scheduled every third month.

In addition to information about healthy eating and recommendations regarding physical activity, the lifestyle support was focused on encouraging parents to be in charge of the treatment outcome. Specific advice was avoided, but the clinical staff could discuss alternatives as well as strategies around conflicts at home around food and eating habits, however the families were encouraged to make their own choices in accordance with motivational interviewing strategies.

Parents and, when deemed suitable adolescents themselves, were instructed to modify eating habits so the BMI *Z*-score remained within the personalized weight target range and act based on deviations from the target. If relative weight loss (BMI *Z*-score decline) was not achieved according to the target curve, the families were encouraged to reflect upon and change eating habits.

The clinical staff and the families corresponded weekly, or whenever the families felt a need for support. Messages were received and sent from the clinic interface for the clinicians and from the mobile application for the families.

### Standard care

The control group received standard pediatric obesity treatment as previously detailed [23]. In short, guidelines for obesity treatment in Sweden include that treatment should be initiated at an early age and before a severe obesity is manifested. Treatment focuses on lifestyle modification to reduce the degree of obesity by improving dietary habits and increasing physical activity in accordance to Nordic recommendations [26]. No pharmacological treatment was available during the study period. Treatment is aimed to be tailored to families’ specific needs and abilities and may therefore be delivered differently.

### Outcome measures

Age was categorized as 4 up to 12 years and from 12 to 18 years at treatment initiation. Degree of obesity was categorized as “obesity” or “severe obesity” according to Cole et al. [25].

The main outcome was the treatment effect on relative weight. This was assessed in three different ways: (1) the change in BMI *Z*-score [25]; (2) the proportion of patients reaching a clinically relevant weight loss (defined as a loss of ≥0.25 BMI *Z*-score units) [23, 27]; and (3) the number of patients in obesity remission.

### Patients receiving digital support

Previous obesity treatment was reported by the referral body and/or from the patient’s own medical file. Data on neuropsychiatric disorders were collected from medical files. Acceptance of use of the device was calculated based on whether the family declined to use the device after information was provided by clinicians during their first clinical visits. Ethnicity data were collected from the parents.

At baseline and every third month, weight was measured at the clinic to the nearest 0.1 kg with participants in light clothing and height was measured to the nearest 0.1 cm without shoes. The weights measured at home as well as the number of measured weights per week during the study period were provided by Evira AB.

### Patients receiving standard care

For the standard care group, weight and height at the start of treatment and at the 1-year follow-up were retrieved from BORIS. Clinics were instructed to measure weight to the nearest 0.1 kg with participants in light clothing and height to the nearest 0.1 cm. The validity of data quality was frequently assessed and has been described previously [23].

### Statistics

Descriptive data are presented as frequencies or mean with standard deviation. The 1-year data at the intervention clinic were defined as 52 ± 2 weeks and for the standard care group 1 year ± 3 months to effectively capture the clinical situation. Attrition rate e.g., missing 1-year follow-up data, were handled with multiple imputation with the predictive mean matching (PMM) method under the assumption that data was missing at random. Factors added to the underlying imputation model were sex, age, and BMI *Z*-score at baseline, and the number of datasets were set to 20. The imputation worked well (Supplementary material, S-Fig. 1). Since different methods can be used to handling missing data, main outcome was also analyzed with baseline value carried forward and an imputation model with other assumptions (Supplementary material).

The main outcome, change in BMI *Z*-scores at 1 year, and other continuous variables were compared between groups using t-test. Differences in proportion was assessed with *χ*^2^ test. A generalized linear model (proc glm) was applied to evaluate the effect of the digital support system adjusted for sex, age, and degree of obesity. Further, analyses for the main outcome were stratified for sex, age group and obesity severity. To assure the representativity of the standard care group, post hoc analyses was performed investigating baseline characteristics, attrition rate and treatment outcome for patients in Stockholm and outside Stockholm County. In the group with digital support, number of clinical visits, message and self-monitoring frequency are based on observed data.

STATA (version 16.0, Stata, College Station, TX) was used for imputation of data and SAS Statistical software (version 9.4, SAS Institute Inc., Cary, NC) was used for the analyses. A *p* value <0.05 was deemed to indicate statistical significance.

### Results

Of 109 consecutively recruited children and adolescents who fulfilled the inclusion criteria, 107 (98%) accepted to use the digital support system. In addition, 321 patients with standard care were included in the 1-year evaluation (Fig. 1). Patient characteristics for both groups are presented in Table 1. In the digital support group, the majority were males (67%) and the age ranged from 4.1 to 17.4 years. 30.8% (*n* = 33) had previously received obesity treatment and the prevalence of neuropsychiatric disorder (e.g. ADHD) was 18.7%. Both groups had similar sex (*p* = 1.0) and age (*p* = 0.93) (matching variables), and BMI *Z*-score at treatment initiation (2.81 (digital support) vs. 2.77 (standard care), *p* = 0.38). Forty-six percent of the children in the intervention group had parents from countries outside Scandinavia and 36% came from countries outside Europe. Information about previous treatment, ethnicity and neuropsychiatric disorders was not available for the standard care group.

**Table 1:** Characteristics of patients.

	Digital support			Standard care		
	Girls	Boys	Total	Girls	Boys	Total
*n* (%)	35 (33)	72 (67)	107 (100)	105 (32.7)	216 (67.3)	321 (100)
Age, mean (min-max)	11.9 (6.4–17.3)	11.9 (4.1–17.4)	11.9 (4.1–17.4)	11.3 (5.6–17.3)	11.3 (3.6–17.2)	11.3 (3.6–17.3)
<12 years, *n* (%)	17 (48.6)	38 (52.8)	55 (51.4)	58 (55.2)	120 (55.6)	178 (55.5)
≥12 years, *n* (%)	18 (51.4)	34 (47.2)	52 (48.6)	47 (44.8)	96 (44.4)	143 (44.6)
Weight, mean (min-max)	71.6 (31.7–119.4)	74.7 (23.9–137.0)	73.7 (23.9–137.0)	64.6 (28.0–114.2)	68.3 (23.0–173.0)	67.0 (23.0–173.0)
Height, mean (min-max)	152.7 (124.0–177.0)	156.6 (110.4–191.0)	155.3 (110.4–191.0)	146.4 (110.1–174.5)	152.7 (103.8–188.0)	150.6 (103.8–188.0)
BMI *Z*-score, mean (min-max)	2.8 (2.2–4.2)	2.8 (2.3–3.8)	2.8 (2.2–4.2)	2.8 (2.20–3.9)	2.8 (2.3–4.1)	2.8 (2.2–4.1)
Severe obesity, *n* (%)	13 (37.1)	25 (34.7)	38 (35.5)	36 (34.3)	60 (27.8)	96 (29.9)
Obesity, *n* (%)	22 (62.8)	47 (65.3)	69 (64.5)	69 (65.7)	156 (72.2)	225 (70.1)
Previous treatment, *n* (%)	11 (31.4)	22 (30.6)	33 (30.8)	Not available	Not available	Not available
Neuropsychiatric disorder, *n* (%)	3 (8.6)	17 (23.6)	20 (18.7)	Not available	Not available	Not available

### Digital support vs. standard care

**Fig. 3:**
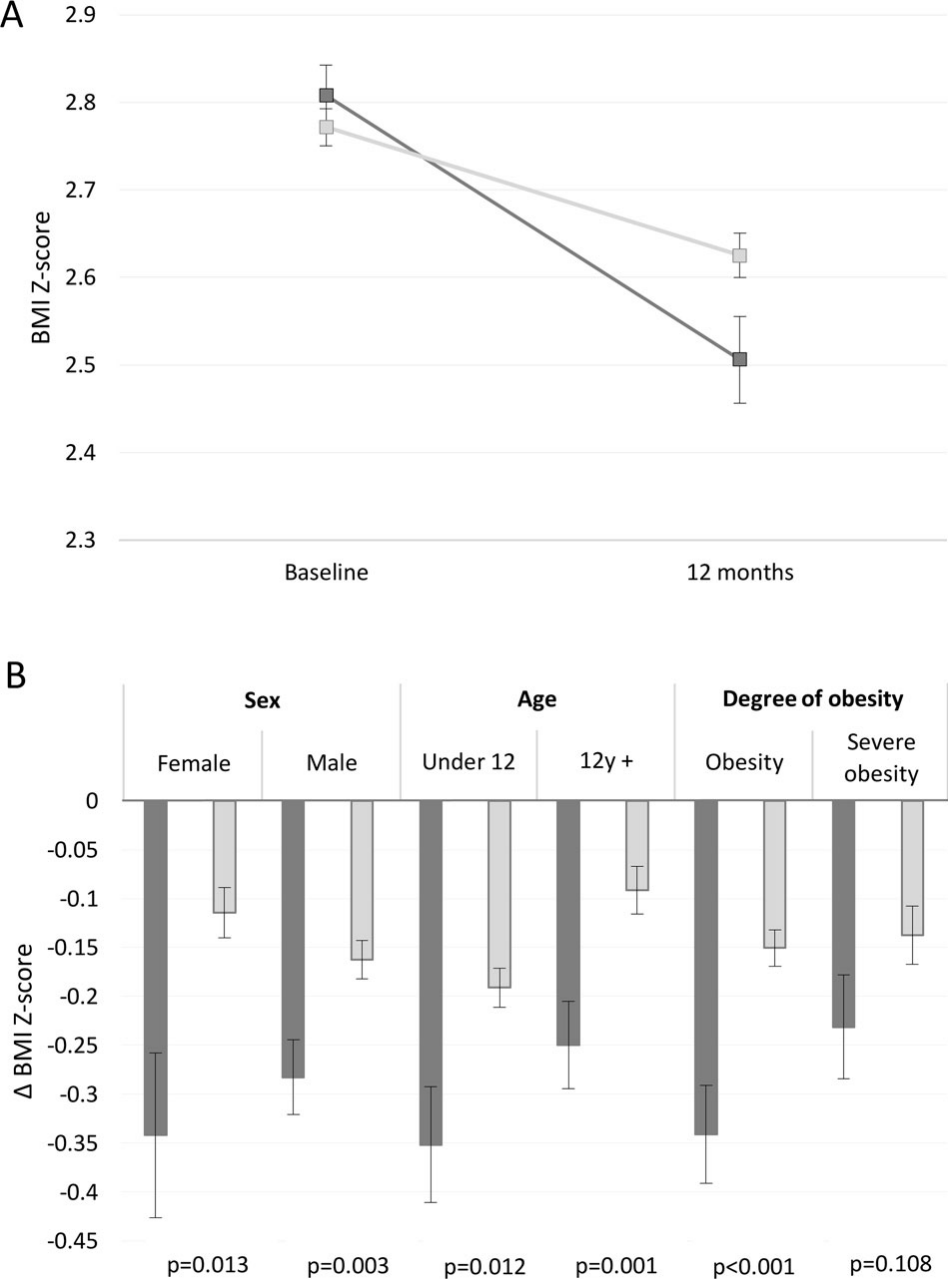
Treatment outcome. Dark gray show outcome for the digital support group and light gray bars indicates standard care. Whiskers indicate standard error. **A** Mean BMI *Z*-score and standard error at baseline and 1 year. **B** Mean change in BMI *Z*-score, 1-year post treatment initiation, stratified for sex, age, and degree of obesity.

The 1-year change in BMI *Z*-score was −0.15 BMI *Z*-score units greater in the digital support group compared with the standard care group (0.30 ± 0.39 vs. 0.15 ± 0.28), complete cases *p* = 0.012 (Supplementary material, S-Fig. 1) and imputed data *p* < 0.001 (Fig. 3a). From here on, only imputed data are presented, unless otherwise stated. Alternative ways of analyzing the outcome, e.g. baseline value carried forward, are presented in Supplementary material, S-Table 1. Regardless of imputation method, digital support yields a better treatment outcome compared to standard care.

In a model adjusted for sex, age, and degree of obesity at treatment initiation, the effect size of the digital support was −0.16 BMI *Z*-score units between the groups, *p* < 0.001. Lower age was associated with 0.10 greater decrease in BMI *Z*-score, while sex and degree of obesity at treatment initiation did not affect the outcome (Table 2).

**Table 2:** Mutually adjusted GLM: Effect size of treatment, adjusted for sex, age and degree of obesity at treatment initiation, *n* = 428.

	Estimate	Standard error	*p*
Digital support vs. Standard care	−0.162	0.035	<0.0001
Female vs. Male	0.018	0.032	0.572
12 + years vs. under 12 years	0.1	0.03	0.001
Severe Obesity vs. Obesity	0.037	0.032	0.251

In stratified analyses, presented in Fig. 3b, the treatment effect was superior among patients with the digital support compared with standard care in both males and females, young children and adolescents, and among those with obesity at baseline. The patients who started treatment with severe obesity had greater 1-year decrease in BMI *Z*-score, however this was not statistically significant (digital support vs. standard care −0.23 ± 0.33 vs. −0.14 ± 0.29, *p* = 0.11).

After 1 year of treatment, 45.8% of patients receiving digital support and 30.5% of those with standard care had a decrease of at least 0.25 BMI *Z*-score units, *p* = 0.004. Among patients with digital support, 25.2% went into obesity remission compared with 17.8% in the standard care group, *p* = 0.09. In order to get an overall assessment of efficacy of treatment, success rate was defined as either obesity remission or a decrease of at least 0.25 BMI *Z*-score units. The success rate among those with digital support was 46.7% compared with 35.5% in the standard care group, *p* = 0.039.

### Attrition rate

**Fig. 4:**
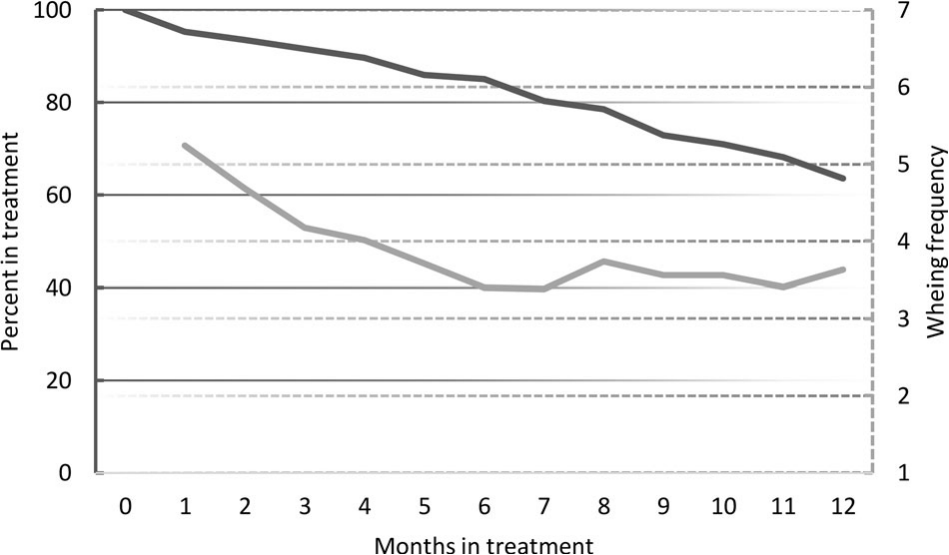
Process measures for individuals receiving digital support. Dark gray line indicates individuals remaining in treatment (left axis) and light gray line show average numbers of weekly weight measurements (right axis).

Attrition rate was 36.4% in the digital support group and 46.1% in the standard care group, *p* = 0.081. In the intervention group, attrition rate was stable over time (Fig. 4). Comparing individuals with and without 1-year data, there were no difference in sex distribution (*p* = 0.92), degree of obesity at start of treatment (*p* = 0.37), neuropsychiatric disorder (*p* = 0.23), or prior treatment (*p* = 0.14). However, individuals in the older age range were more likely to be lost to follow-up (*p* = 0.015). In standard care, the lost to follow-up did not differ by sex (*p* = 0.07), age (*p* = 0.55) or degree of obesity at the start of treatment (*p* = 0.15).

### Extended standard care group analyses

The standard care individuals were randomly selected from all areas of Sweden whereas the digi-physical group was from the Stockholm County. The treatment outcome may be affected by living conditions and thereby the comparison between the two intervention groups. Of the 321 individuals in the standard care group, 58 (18%) were from the Stockholm County. Compared with patients outside Stockholm (*n* = 263), the patients in Stockholm had similar sex distribution (64% vs. 68% males, *p* = 0.53), age (11.3 vs. 11.3 years, *p* = 0.90), BMI (28.3 vs. 28.51 kg/m^2^, *p* = 0.81), and BMI *Z*-score (2.73 vs. 2.78, *p* = 0.36) at baseline. The attrition rate among patients from Stockholm was higher compared with patients from other parts of the country, 60.3% vs. 43.0%, *p* = 0.016. With observed data between patients in Stockholm (*n* = 23) had an average change of −0.12 *Z*-score units and patients outside Stockholm (*n* = 150) had −0.14 *Z*-score units, *p* = 0.74. Analyses with imputed data shows a change of −0.16 *Z*-score units among the Stockholm patients and 0.14 *Z*-score units among patients outside Stockholm, *p* = 0.75.

### Outcomes in individuals with digital support

#### Weekly treatment outcome

The continuous change in BMI *Z*-score for observed data over 1 year is illustrated in Supplementary material, S-Fig. 2. After ~9 months an average decrease of 0.25 BMI *Z*-score units was observed. One-year treatment outcome (imputed data) did not differ between sexes (*p* = 0.52), age groups (*p* = 0.17), degree of obesity (*p* = 0.16), or presence of neuropsychiatric disorder (*p* = 0.93). The only identified factor significantly affecting the outcome was if the child had received previous obesity treatment, where those who had not received previous treatment decreased their BMI *Z*-score more (−0.35 ± 0.45 vs. −0.20 ± 0.23, *p* = 0.03).

#### Number of clinical visits and messages

The number of physical clinical visits, including initiation of treatment, range from 2 to 14 with a median of 6 visits. The median (IQR) of messages sent from the clinic to the patient was 42(21.5) and the number of messages from patients to the clinic was 19 [21].

#### Self-monitoring frequency

The average frequency of daily weight measurements was highest in the beginning of the treatment, with more than five weight measurements per person and week during the first month, later stabilizing at 3.7 ± 2.2 weight measurements per person and week for the remaining year, Fig. 4.

No harms of treatment (such as depression or eating disorders) were reported.

## Discussion

In this pragmatical clinical trial, we investigated the effect of pediatric obesity lifestyle treatment facilitated by a digital support system compared to randomly selected matched controls from a pediatric obesity treatment register. The 1-year treatment results were superior compared with the conventionally treated standard care group. The mean relative weight loss was twice as large in those who received digital support (−0.30 vs. −0.15 BMI *Z*-score units) and a greater proportion of patients obtained a clinically significant weight loss, defined as a decline of 0.25 BMI *Z*-score units (45% vs. 30%).

Even though, the study was not powered for sub-analyses, and they therefore should be interpreted with caution, the findings indicate that digital support may be more favorable in some groups. The most remarkable difference between the groups was observed among adolescents with 2.7 times better outcome, which was beyond our expectations since we and others have failed to effectively treat this age group [27, 28].

The effect of treatment with digital support was large compared with previously published results of childhood obesity treatment. An average reduction of 0.13 BMI *Z*-score units was found in an overview of six Cochrane reviews of childhood obesity treatment [8], which is similar to the effect seen in our standard care group (−0.15 units). In a review by the US Preventive Services Task Force, it was concluded that the frequency of physical visits was the most important factor for treatment success and a reduction of 0.17 BMI *Z*-score units was obtained after at least 26 visits in 1 year [9]. In the present study, individuals with digital support reached a weight loss of 0.30 BMI *Z*-score units with a median of six physical visits in 1 year. Thus, we conclude that it is possible to replace physical visits with this type of digital support system.

There are several features of the treatment program that may have contributed to the strong results. Daily weight measurements have been shown to be of importance for adults [11, 12]. The interpretation and graphical presentation of BMI *Z*-score enables the families to follow the progression of obesity treatment. This, in turn, provides the families with a tool that helps them to be in control of the treatment. The weighing frequency remained high for a majority of the children throughout the treatment year. The digital contact with the clinical staff was focused on support and education rather than advice. Thus, these aspects of the program resemble the motivational interviewing technique.

As body weights are presented to the clinic continuously, support can be provided as soon as there are signs of failure of adherence to treatment or the set goals are not met. Hence, weight regain, which is common in all types of treatment [29, 30], may be prevented to a large extent and a continuous relative weight loss could be achieved during the entire treatment year (Supplementary Fig. 2).

Thus, we believe that the main contributions from the digital support system are twofold. Firstly, it gives the patients and their families a fast tangible feedback loop. Secondly, it contributes to an efficient communication, enabling the medical expertise to focus their efforts in the right moment to the right patients.

The attrition rate was 10 percent units lower among individuals with digital support compared to standard care (36.4% vs. 46.1%), a difference which was not statistically significant. However, the children who received digital support were all from Stockholm County and the attrition rate was even higher among the children with standard care from Stockholm County (60%). An important factor for attending clinical visits is treatment satisfaction but logistic barriers, such as travel distance or school and work absence may contribute to attrition [31]. Another explanation could be reluctance to attend the clinic due to weak treatment achievement. The families who struggled with poor weight development knew in advance that the clinical staff were aware of their problems which may have reduced the negative expectations before the visits. We believe that as the visits are focused on how to overcome current problems instead of discussing past failures, the late cancellations and dropouts may be reduced, as was observed in our previous randomized feasibility study [22]. Finally, the low frequency of physical visits enabled families that live far from the clinic to remain in the program.

More potent treatments are often associated with a higher risk of unfavorable side effects. We did not observe any harm of treatment, e.g. eating disorders, in this study. In adults, daily weight measurements are not associated with an increased risk of eating disorders [10] and conventional childhood lifestyle obesity treatment is associated with a reduced risk of eating disorders [32]. Monitoring daily weight measurements in patients in obesity treatment enables early identification of abnormal weight changes and weighing patterns.

There are several strengths of this 1-year pragmatical trial. The random selection of a large group of control individuals from several pediatric clinics made it possible to compare the investigated treatment with the present real-life clinical situation. Using register data allows a larger sample and longer duration of follow-up. Further, pragmatic clinical trials reduce potential disappointment bias of being randomized to unwanted treatment, which can negatively affect the motivation for lifestyle changes and thereby may affect the results of treatment. In addition, using clinical register data lowers the Hawthorne effect, i.e. that individuals (both patients and clinical staff) modify their behavior in response to their awareness of being observed. Taken together, these aspects confirm that the external validity of this study is relatively high. The situation is more complicated in non-blinded randomized controlled trials (RCT). Disappointment bias may negatively affect compliance and outcome for those who chose to participate in a trial where a new type of treatment is tested but are randomized to a conventional treatment. This is a confounder in non-blinded RCTs which is not sufficiently considered.

There are also limitations that should be acknowledged. We lack data on psychological health and number of clinical visits for individuals in the standard care group. However, we can conclude that the control group consist of a representative sample of children in Sweden that receive standard obesity treatment [23]. It is worth mentioning that the treatment outcome in the standard treatment care group is relatively good compared to other studies [7]. The group that received digital support was from Stockholm County. To study if the outcome in general was better for children living in Stockholm, we conducted a post hoc analysis of the patients from Stockholm County in the standard care group and compared their outcome with the rest of Sweden. The treatment results were similar, but as mentioned, the attrition rate was significantly higher among patients in Stockholm.

The investigated digital support was evaluated in one single obesity clinic, a standard open pediatric ward, and the clinic did not have extra resources except for the digital system. However, as always when chronic diseases are treated, the combination of optimized technical support and a dedicated treatment staff is required to improve outcome and it remains to be established how different clinics and cultural settings affect treatment outcome. Another study design is also required to evaluate cost-effectiveness and generalizability. Consequently, an international randomized multi-center study is therefore of major importance to confirm the present results.

Finally, the non-randomized design reduces the internal validity. However, as mentioned above, many factors contribute to an improved external validity which is in line with that pragmatical clinical trials in general have higher external validity than classic clinical trials [24].

The longevity of digital obesity treatment remains to be evaluated. However, the present digital system encourages a stable weight loss, which may increase the longevity of treatment effect. The personalized medicine is strengthened by the individualized treatment goals. The individualized target curves, i.e. the personalized medicine, emphasizes the empowerment of the families through active participation in managing the health of their children, which is of great importance for the longevity of treatment.

## Conclusion

Our findings indicates that the 1-year outcome of a childhood obesity lifestyle treatment program with the combination of physical visits and an interactive digital support system including objective data is superior to standard lifestyle treatment.

## Supplementary information

Supplementary material

Anonymized dataset

Checklist

## Data Availability

The datasets generated during and/or analyzed during the current study are available as Supplementary material. Additional data can be retrieved from the corresponding author on reasonable request.
