# Supplementary material for: Effect of an interactive mobile health support system and daily weight measurements for pediatric obesity treatment, a 1-year pragmatical clinical trial
Source: Int J Obes (Lond). 2022 May 31;46(8):1527–33. doi: 10.1038/s41366-022-01146-8 (PMC9314258; doi:10.1038/s41366-022-01146-8)
Supplement: Supplementary file 1 — Supplementary material [file 41366_2022_1146_MOESM1_ESM.docx]

# Observed vs. imputed data


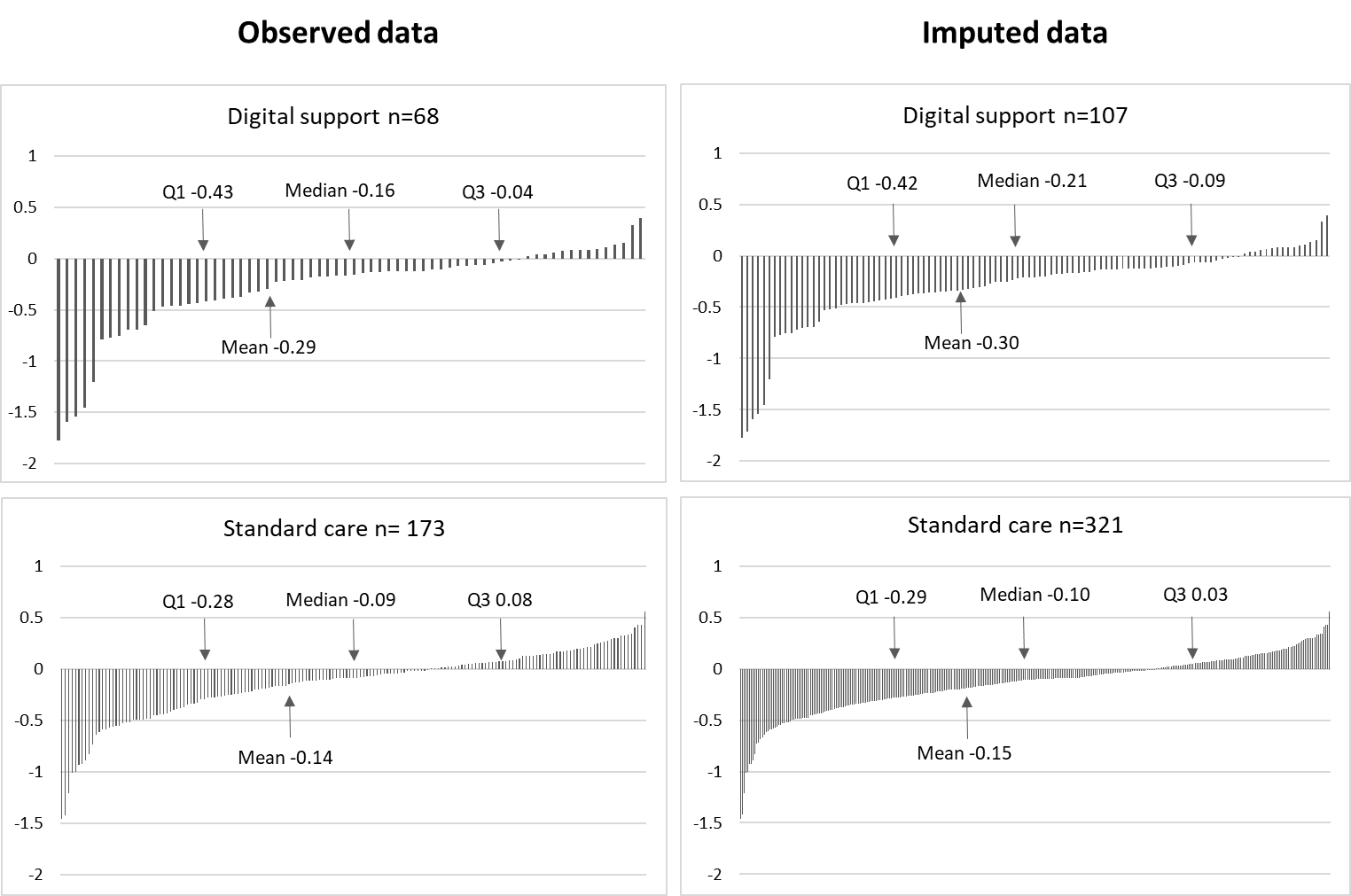


Supplementary Figure 1. Individually achieved change in BMI Z-score, one-year post treatment initiation, for digital support group and standard care. Complete cases vs. imputed data.

# Several ways to analyze main outcome

Since different methods can be used to handling missing data, two additional statistical analyses have been performed. One of them use Baseline Observation Carried Forward (BOCF), assuming that those without one-year data have remained at their original degree of obesity. The other method is a model where all missing data were handled with the multiple imputation method under the assumption that patients who did not have a one-year follow-up responded as though they had been treated with standard care for the entire trial. Analyses are adjusted for sex, age and degree of obesity at treatment initiation.

| Supplementary Table 1. Analyses of change in BMI Z-score at one year using different methods of handling missing data | | | |
| --- | --- | --- | --- |
|  | Digital support  n=107 | Standard Care  n=321 |  |
|  | Mean (standard deviation) | Mean (standard deviation) | p-value |
| Observed data (in article) | -0.29 (0.43) | -0.14 (0.34) | 0.012 |
| Ordinary imputation (in article) | -0.30 (0.39) | -0.15 (0.28) | 0.0002 |
| Baseline Observation Carried Forward | -0.18 (0.37) | -0.07 (0.26) | 0.0057 |
| Imputation based on standard care outcomes | -0.20 (0.38) | -0.11 (0.27) | 0.019 |

# Average cumulative change in BMI Z-score


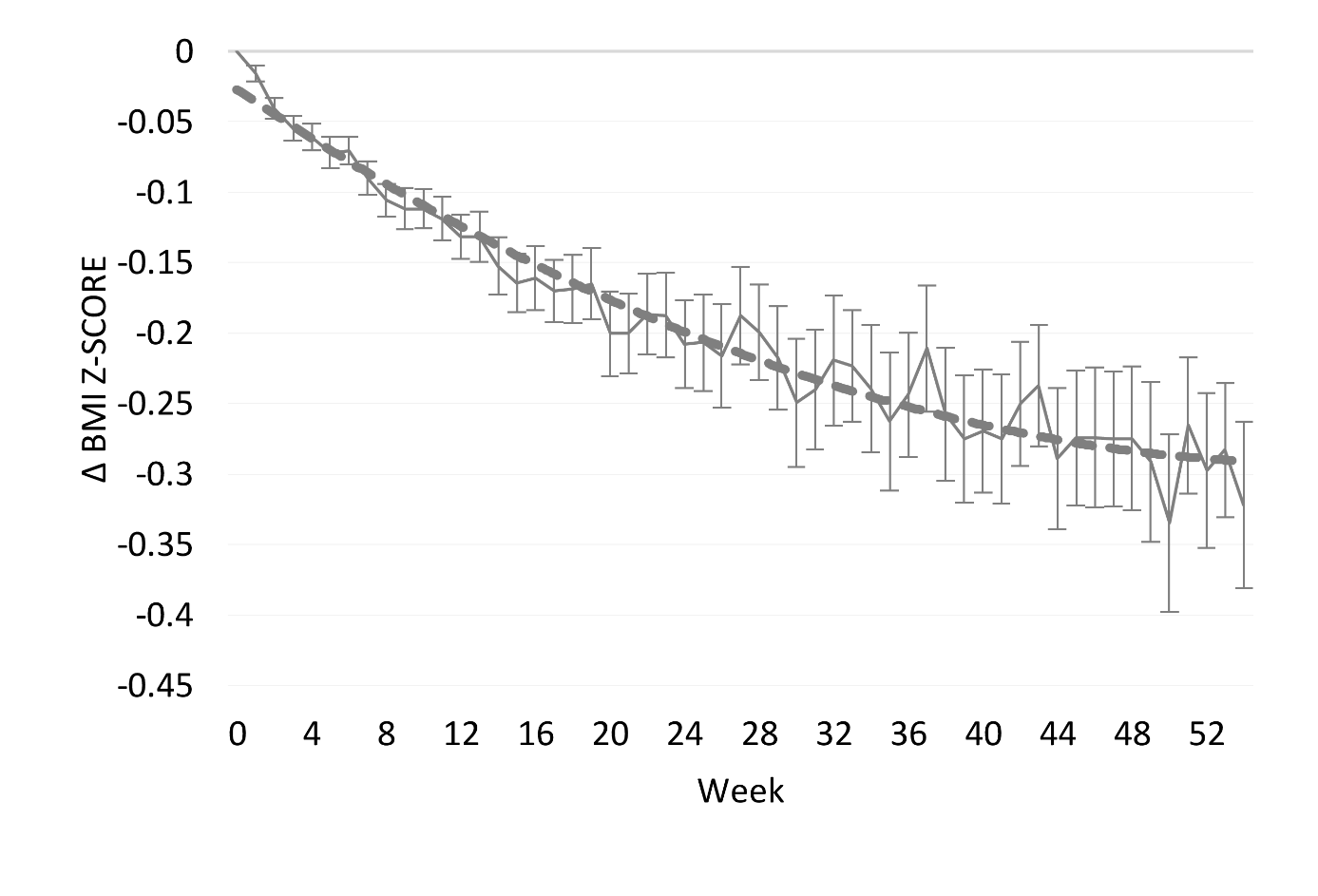


Supplementary Figure 2. Observed weekly average change in BMI Z-score. Error bars present standard error.

# Height estimations

The longitudinal growth was estimated by the digital system in between the height measurements at the clinic. Since children with obesity have an altered height growth compared with normal weight peers, an obesity-specific predictive longitudinal growth estimation algorithm was constructed taking into consideration, current height, degree of obesity, age and sex. These estimations were generated from an adaptive nearest neighbor regression model fitted on data from the BORIS registry[1].

The accuracy of height estimation was investigated based on 288 follow-up visits in 83 whom have not reached final height. Overall, the median measured height was 0.16cm (q1: -0.46, q3: 1.01) greater. The difference between estimated and measured height was similar for both sexes (p=0.27), BMI Z-score at baseline (p=0.45), and absolute height at baseline (p=0.22). However, the difference was 0.05cm smaller for every year older the individuals were (p=0.048).

1 Hagman E, Danielsson P, Lindberg L, Marcus C, Committee BS. Paediatric obesity treatment during 14 years in Sweden: Lessons from the Swedish Childhood Obesity Treatment Register-BORIS. *Pediatr Obes* 2020; **15:** e12626.
